# Nimble Cloning: A Simple, Versatile, and Efficient System for Standardized Molecular Cloning

**DOI:** 10.3389/fbioe.2019.00460

**Published:** 2020-01-15

**Authors:** Pu Yan, Yanjing Zeng, Wentao Shen, Decai Tuo, Xiaoying Li, Peng Zhou

**Affiliations:** Key Laboratory of Biology and Genetic Resources of Tropical Crops, Ministry of Agriculture, Institute of Tropical Bioscience and Biotechnology, Chinese Academy of Tropical Agricultural Sciences, Haikou, China

**Keywords:** molecular cloning, standardized cloning, Gibson assembly-derived cloning, vector construction, DNA assembly, prokaryotic expression, plant gene expression

## Abstract

Molecular cloning is one of the most fundamental technologies in molecular biology, and has been critical for driving biotechnological advances. In this study, we have developed a novel method for standardized molecular cloning. The cloning technique known as “Nimble Cloning” uses the restriction enzyme, *Sfi*I, in combination with the T5 exonuclease, to linearize the vector and generate 3′-overhangs simultaneously. Both PCR products and plasmids can be used for the cloning reaction in the Nimble Cloning system. The cloning system is highly efficient, suitable for gene expression in both prokaryotic and eukaryotic expression systems, and enables the reuse of DNA fragments or plasmid entry clones. Nimble Cloning is applicable for the cloning of single or multiple fragments, as well as multi-site cloning. Due also to its simplicity and versatility, the cloning method has great potential for the modular assembly of DNA constructs.

## Introduction

Molecular cloning, which is one of the most fundamental procedures available for modern molecular biology research, has been critical for driving biotechnological advances. One of the main objectives in the post-genomics era is to functionally map gene expression data. Thus, developing methods for the rapid and efficient construction of various vectors for transgenic research is more critical now than ever before.

To the best of our knowledge, of the many molecular cloning protocols that have been developed, the following are the main techniques currently used for routine cloning: restriction digestion- and ligation-based cloning (Cohen et al., 1973), Gateway cloning (Hartley et al., 2000), Gibson assembly (Gibson et al., 2009), and Golden Gate cloning (Engler et al., 2008). Each of these methods have specific limitations (Liang et al., 2017). Traditionally, type II restriction endonucleases and DNA ligases have been used to construct recombinant plasmids, and these enzymes are still extensively used for diverse molecular biology applications. However, this method is laborious and time-consuming and is often limited by the relatively few available restriction enzyme sites, especially during the assembly of complex plasmids from multiple elements (Lampropoulos et al., 2013; Wang et al., 2014). Gateway cloning is a very popular site-specific recombination system that exploits the lambda phage integration and excision mechanism (Hartley et al., 2000). Although Gateway cloning has been widely used in many experimental systems, the following are the four main disadvantages with this method: (i) the associated two-step (BP and LR recombination reactions) cloning process is labor-intensive and time-consuming; (ii) the recombination site leaves a 25-bp unwanted junk sequence (scar); (iii) the assembly of multiple fragments is relatively inefficient; and (iv) the commercial enzyme mixes available for this method are expensive, especially for laboratories in developing countries. As an alternative, Gibson assembly is a quick and easy method that enables the linking of multiple overlapping DNA fragments in a single isothermal reaction (Gibson et al., 2009). The recombination is based on the homologous ends (15–20 bp). Other *in vitro* recombination methods may involve the same homologous ends, including the In-Fusion (Zhu et al., 2007; Sleight et al., 2010), Gibson-derived Hot Fusion (Fu et al., 2014), and TEDA (Xia et al., 2018) procedures. These methods are mostly sequence-independent, and because of this sequence flexibility, there is currently no standard protocol for designing the overlapping sequences. Additionally, Gibson assembly requires a destination vector that is linearized by enzyme digestion or PCR, making it a method that is not totally free of restriction enzymes. Golden Gate cloning relies on the type IIs restriction enzymes, and is capable of assembling numerous fragments with high efficiency and fidelity (Engler et al., 2009; Engler and Marillonnet, 2011), but the DNA fragment to be assembled needs to be free of the recognition sequence of the enzymes used. Type IIs restriction sites are often shorter than 7 bp and are frequently present within DNA sequences to be cloned, thereby limiting the application of Golden Gate cloning, especially for the cloning of long DNA fragments and multiple DNA fragments.

In this study, we have developed a novel method for standardized molecular cloning known as Nimble Cloning. This method, which is based on the Gibson assembly technique (Gibson et al., 2009), requires a simple enzyme mix of the restriction enzyme, *Sfi*I, and T5 exonuclease. These two enzymes simultaneously mediate the linearization of the plasmid vector and the generation of 3′-overhangs of the insert DNA fragments. We demonstrate that this cloning method is simple and efficient, and has great potential as a versatile tool for assembling DNA constructs.

## Materials and Methods

### Strains, Reagents, and Cell Cultivation

*Escherichia coli* strains DH5α and DB3.1 (Transgen Biotech, Beijing, China) were used for cloning. *Agrobacterium tumefaciens* strain GV3101 was used for transforming plants. The *E. coli* cultures were grown at 37°C in Lysogeny Broth (LB) selection medium. The *Sfi*I, T5 exonuclease, and Phusion polymerase enzymes as well as the Gibson Mix were obtained from New England Biolabs (Ipswich, MA, USA). Primers were ordered from ThermoFisher (Guangzhou, China).

### Plasmid Construction

The destination and entry vectors of the Nimble Cloning system were generated by inserting their cloning cassettes into the desired vector via Gibson assembly. The cloning cassette for the destination vector was “adapter 1–*Sfi*I site–*ccdB* gene–*Sfi*I site–adapter 2” (NC frame), whereas that for the entry vector was “*Sfi*I site–adapter 1–XcmI–*ccdB* gene–XcmI–adapter 2–*Sfi*I site.” Details regarding the primers used to construct these vectors are listed in Supplementary Table S1. The “*Sfi*I–adapter 1–XcmI–*ccdB* gene–XcmI–adapter 2–*Sfi*I” fragment was inserted into the cloning sites of the pENTR/D-TOPO (Invitrogen) and pMD-18T (Takara, Dalian, China) vectors to produce the entry vectors with kanamycin and ampicillin resistance genes, respectively.

The pNC-UC destination vector was constructed by replacing the *lacZ* sequence of pUC19 with the NC frame. The prokaryotic expression vector pNC-ET28 was constructed by inserting the NC frame into pET28 between the NdeI and XhoI sites. The plant expression vector pNC-Cam1304 was constructed by inserting the NC frame into pCAMBIA1304 between the NcoI and PmlI sites, which replaced the GFP/GUS sequences. The pNC-Green vector was constructed by inserting the NC frame into pGreenII 0000 (Hellens et al., 2000) between the HindIII and EcoRI sites. The NC-frame and GFP, and GFP and NC-frame fragments were cloned into pGreenII-35S (Tuo et al., 2017) between the 35S promoter and the CaMV polyA terminator to generate the plant sublocation vectors pNC-GFP-C and pNC-GFP-N, respectively. The plant RNAi vector pNC-RNAi was constructed by inserting the NC frame, Pdk intron (Yan et al., 2012), and the inverted NC frame into pCAMBIA1304 between the NcoI and PmlI sites. To construct the double open reading frame (ORF) expression BiFC vector pNC-BiFC, the NC frame was first inserted into pSAT-nEYFP-N1 and pSAT-cEYFP-N1 (Citovsky et al., 2006) between BglII and BamHI. The two expression cassettes, including the promoter and the terminator, were then amplified and cloned into pGreen 0029 (Hellens et al., 2000) between the HindIII and EcoRI sites. The maps of the destination vectors were listed in Supplementary Figure S1.

### Preparation of Nimble Mix

A 500-μl sample of 2× Nimble Mix was prepared by mixing 200 μl 5× Nimble buffer (25% PEG-8000, 0.5 M Tris, pH 7.5, 50 mM MgCl_2_, and 50 mM DTT), 4 μl T5 exonuclease (1 U/μl), 40 μl *Sfi*I (2 U/μl), and 256 μl ddH_2_O. The Nimble Mix was divided into 50–100 μl aliquots in 1.5-ml microcentrifuge tubes and stored at −20°C.

### Nimble Cloning Reaction

We added 20–100 ng circular destination vector (1–2 μl) and 10–50 ng PCR insert or entry clone plasmid (1–3 μl) to a PCR microtube containing 5 μl 2× Nimble Mix, after which distilled water was added to the tube for a final volume of 10 μl. Multiple fragments were assembled with 1 μl each insert (10–30 ng). Tubes were incubated in a water bath or a thermocycler for 1 h at 50°C, and the reaction mixture was subsequently used for a transformation or stored at −20°C if not immediately used (Supplementary Methods S1).

### *Escherichia coli* Transformation

We added 2 μl Nimble reaction mixture to 50 μl *E. coli* DH5α competent cells in tubes, which were then incubated on ice for 30 min, heated at 42°C in a water bath for 45 s, and cooled on ice for 2 min. Next, 450 μl LB medium was added to the tubes, which were then incubated at 37°C for 1 h, with shaking at 200 rpm. An 80-μl aliquot of each cell suspension was spread evenly on agar-solidified LB medium supplemented with specific antibiotics for screening.

### *Nicotiana benthamiana* Transient Expression

Wild-type *Nicotiana benthamiana* was used to analyze transient expression following an agroinfiltration step that was completed according to a slightly modified version of a published procedure (Sparkes et al., 2006, Yan et al., 2012). Plasmids were inserted into *A. tumefaciens* strain GV3101 cells by electroporation. A single colony was then used to inoculate 5 mL YEP medium (10 g/l Bacto-Tryptone, 10 g/l yeast extract, and 5 g/l NaCl, pH 7.0) supplemented with 50 mg/l rifampicin and 50 mg/l kanamycin. Bacteria were grown overnight at 28°C, with shaking at 200 rpm, for an optical density at 600 nm (OD_600_) of 1.0–1.5. The cultures were centrifuged at 2,000 × g for 5 min, after which the pelleted cells were diluted with infiltration buffer (50 mM MES, pH 5.6, 10 mM MgCl_2_, and 100 mM acetosyringone) for a final OD_600_ of 0.2–0.4. The cell solutions were incubated for 1–2 h at 25°C in darkness before being used for an agroinfiltration of *N. benthamiana* leaves with a 1-ml needleless syringe. The GFP fluorescence was observed with a FluoView FV1000 confocal microscope (Olympus, Japan) or a UV lamp (UVP, Upland, CA, USA).

## Results

### Design of the Nimble Cloning System

**Figure 1:**
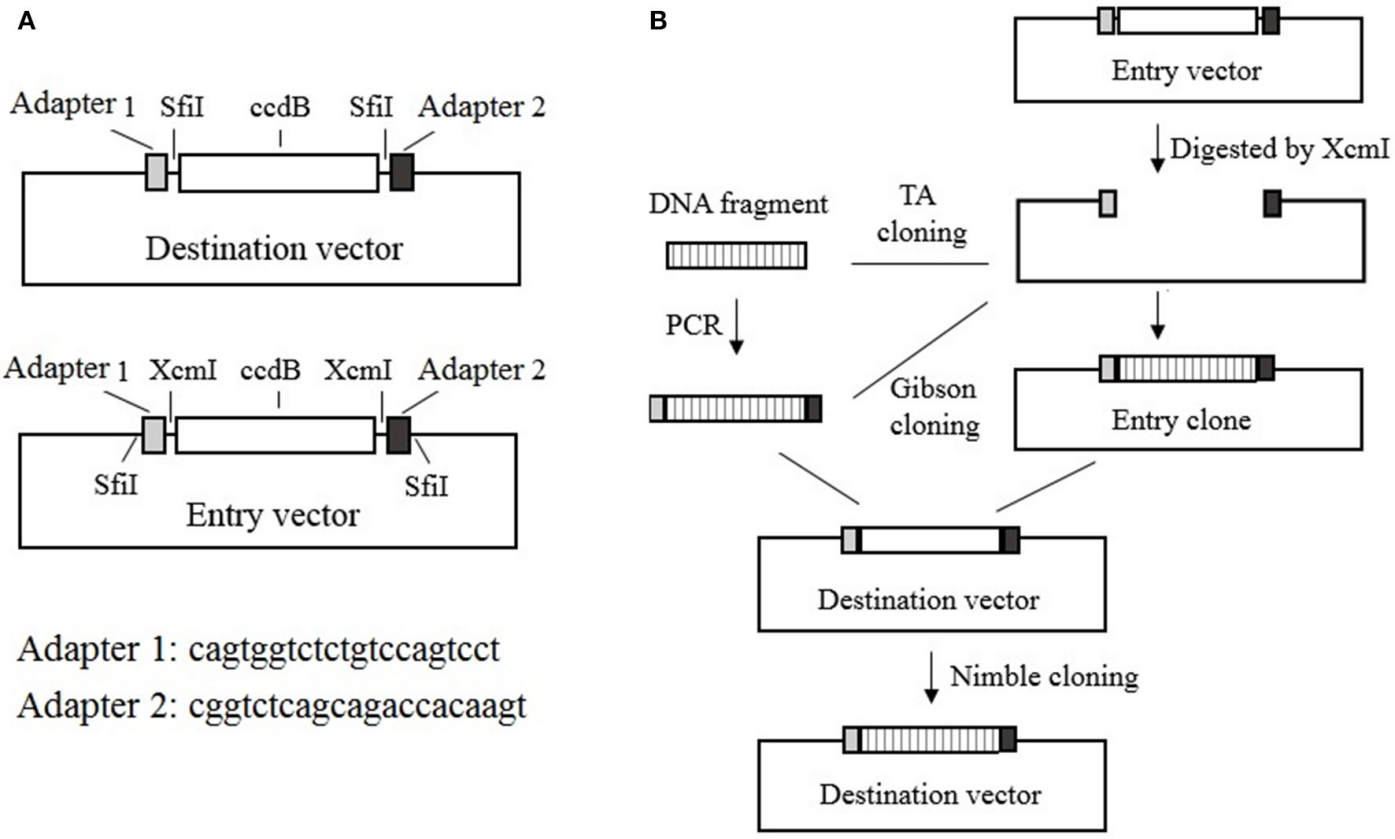
Nimble Cloning system. **(A)** Schematic of the destination and entry vectors in the Nimble Cloning system. The destination vector contains the NC frame comprising the “adapter 1–*Sfi*I–*ccdB* gene–*Sfi*I–adapter 2” sequence, whereas the entry vector contains the “*Sfi*I–adapter 1–XcmI–*ccdB* gene–XcmI–adapter 2–*Sfi*I” sequence at the cloning site. **(B)** Schematic of the Nimble Cloning method. The PCR product flanked by the adapters or the DNA fragment in the entry clone can be cloned into the circular destination vector in a one-step Nimble Cloning reaction. The DNA fragment can be inserted into the entry vector to form the entry clone via TA cloning, or by Gibson cloning using the adapters as overlapping sequences, after the entry vector is digested with XcmI. Nimble Cloning involves *Sfi*I and T5 exonuclease.

The Nimble Cloning system includes recipient expression (destination) vectors and entry vectors. All of the destination vectors contain the NC frame, which comprises the “adapter 1–*Sfi*I–*ccdB* gene–*Sfi*I–adapter 2” sequence in the recombination site. In contrast, the entry vectors contain the “*Sfi*I–adapter 1–XcmI–*ccdB* gene–XcmI–adapter 2–*Sfi*I” sequence in the cloning site, with kanamycin or ampicillin resistance genes. During a Nimble Cloning reaction, the entry clone should carry a different resistance gene from that in the destination vector. A DNA fragment can be inserted into the entry vector to form the entry clone via TA cloning, or by Gibson cloning with adapters as overlapping sequences, after the entry vector has been digested with XcmI. The DNA fragment in the entry clone or the PCR product flanked by the adapters can be cloned into the circular destination vector with the Nimble Mix during the Nimble Cloning reaction and the transformation of *E coli*. The Nimble Mix consists of two enzymes, *Sfi*I and T5 exonuclease (Figure 1).

The two unique 21-bp nucleotide sequences that form the NC frame adapters required for recombination were designed based on the criteria to increase the likelihood of accurate assembly and minimize the possibility that the expression levels of the nearby genes are affected (Torella et al., 2014a). Thus, we computationally designed adapters 1 and 2 comprising the cagtggtctctgtccagtcct and cggtctcagcagaccacaagt sequences, respectively, and used them for the tests of cloning efficiency and gene expression. These two adapters enable efficient assembly and are appropriate for gene expression, as confirmed in the subsequent subsections.

### Cloning of a Single Gene Fragment and Assembly of Multiple DNA Fragments With Nimble Cloning

**Figure 2:**
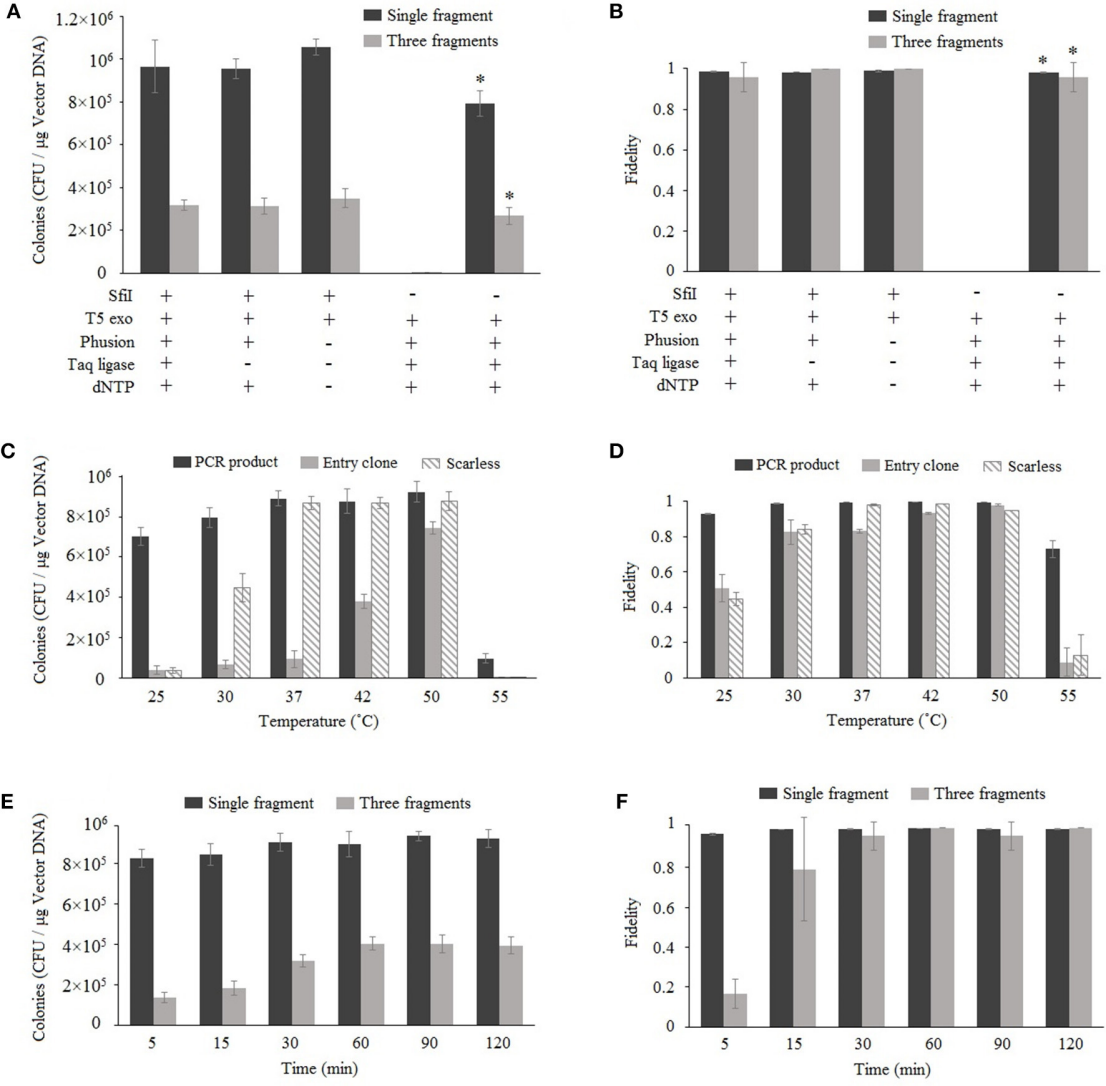
Optimization of Nimble Cloning. Effects of enzymes and buffer components **(A,B)**, reaction temperatures **(C,D)**, and reaction times **(E,F)** on the efficiency and fidelity. The pNC-UC vector was used as the destination vector in each reaction. The *lacZ* fragment was used for the cloning of a single fragment, whereas the 35S promotor, GFP gene, and Tnos fragments were used for the cloning of multiple fragments **(A,B,E,F)**. The PCR product and the entry clone with *lacZ* as well as the *lacZ* PCR product without the unique adapter sequences (scarless) were used in **(C,D)**. ^*^Gibson assembly with the linearized pNC-UC vector. Fidelity was analyzed based on the ratio of blue colonies to total colonies for the cloning of a single fragment, and the ratio of PCR-positive colonies to tested colonies (*n* = 24) for the cloning of multiple fragments. Data are presented as the average (and standard deviation) of three parallel experiments.

Nimble Cloning is based on Gibson assembly, and both methods involve the enzyme-catalyzed assembly of overlapping DNA fragments. Gibson assembly generally applies three enzymes, namely T5 exonuclease, Phusion DNA polymerase, and Taq DNA ligase; however, Taq DNA ligase can be removed without decreasing the cloning efficiency (Fu et al., 2014; Benoit et al., 2016). The more recently developed TEDA method, which requires only T5 exonuclease, results in efficient cloning (Xia et al., 2018). We assessed the enzyme requirements for Nimble Cloning reactions involving one or multiple DNA fragments. The pNC-UC (20 ng) vector used as the destination vector in each reaction was derived from pUC19, with the NC frame replacing the *lacZ* sequence. The *lacZ* gene (10 ng) as well as the 35S promotor, GFP gene, and nos termination sequence (Tnos) (10 ng) were used for the cloning of single and multiple DNA fragments, respectively (Figures 2A,B). For multiple-fragment cloning, the junctions of the internal fragment do not use the unique adapters, but fragment-specific sequences (Supplementary Table S1). The reaction was completed at 50°C for 1 h. The results indicated that Nimble Cloning with *Sfi*I and T5 exonuclease worked well for the cloning of single and multiple DNA fragments. Moreover, the cloning efficiency was higher than that of Gibson assembly, which involves the linearized pNC-UC vector and three enzymes. All reactions for the cloning of *lacZ* yielded >99% positive clones (i.e., blue colonies) on LB plates supplemented with X-gal and IPTG. For the cloning of three fragments, 24 colonies in each group were tested by colony PCR and > 95% had the correct band. Twelve colonies resulting from the Nimble Cloning of single and multiple DNA fragments were further analyzed by sequencing, and no errors were detected.

### Optimal Reaction Temperature and Duration for Nimble Cloning

To determine the optimal reaction temperature, six different temperatures (25, 30, 37, 42, 55, and 55°C) were tested for the standardized cloning (with unique adapters for the PCR product and entry clone) and scarless cloning (without unique adapters) methods (Figures 2C,D). The results following a 1-h reaction revealed that the 37–50°C range was appropriate for the standardized cloning with a PCR product and the scarless cloning. However, 50°C was optimal for the standardized cloning with an entry clone.

Six different time points (5, 15, 30, 60, 90, and 120 min) were tested for the cloning of one or three fragments (Figures 2E,F) at 50°C. The data indicated that the efficiency and fidelity of the cloning of a single fragment were high even for the shortest time point (5 min), and the efficiency plateaued after 30 min. Regarding the cloning of three fragments, the efficiency and fidelity increased over time and plateaued after 60 min. Three PCR-negative colonies in the three-fragment Nimble Cloning with the reaction time of 5 min were sequenced, and they all were the products of self-ligation of the vector between the two *Sfi*I sties of NC frame with the overlap sequences “GGCC.”

### Application of Nimble Cloning for Prokaryotic and Plant Expression Systems

Unique adapters are used in the Nimble Cloning system for standardized cloning reactions. To confirm that unique adapters do not adversely affect gene expression, we evaluated the expression of sequences from the plasmids constructed by standardized cloning (with unique adapters) and scarless cloning (without unique adapters) in both prokaryotic and plant expression systems.

**Figure 3:**
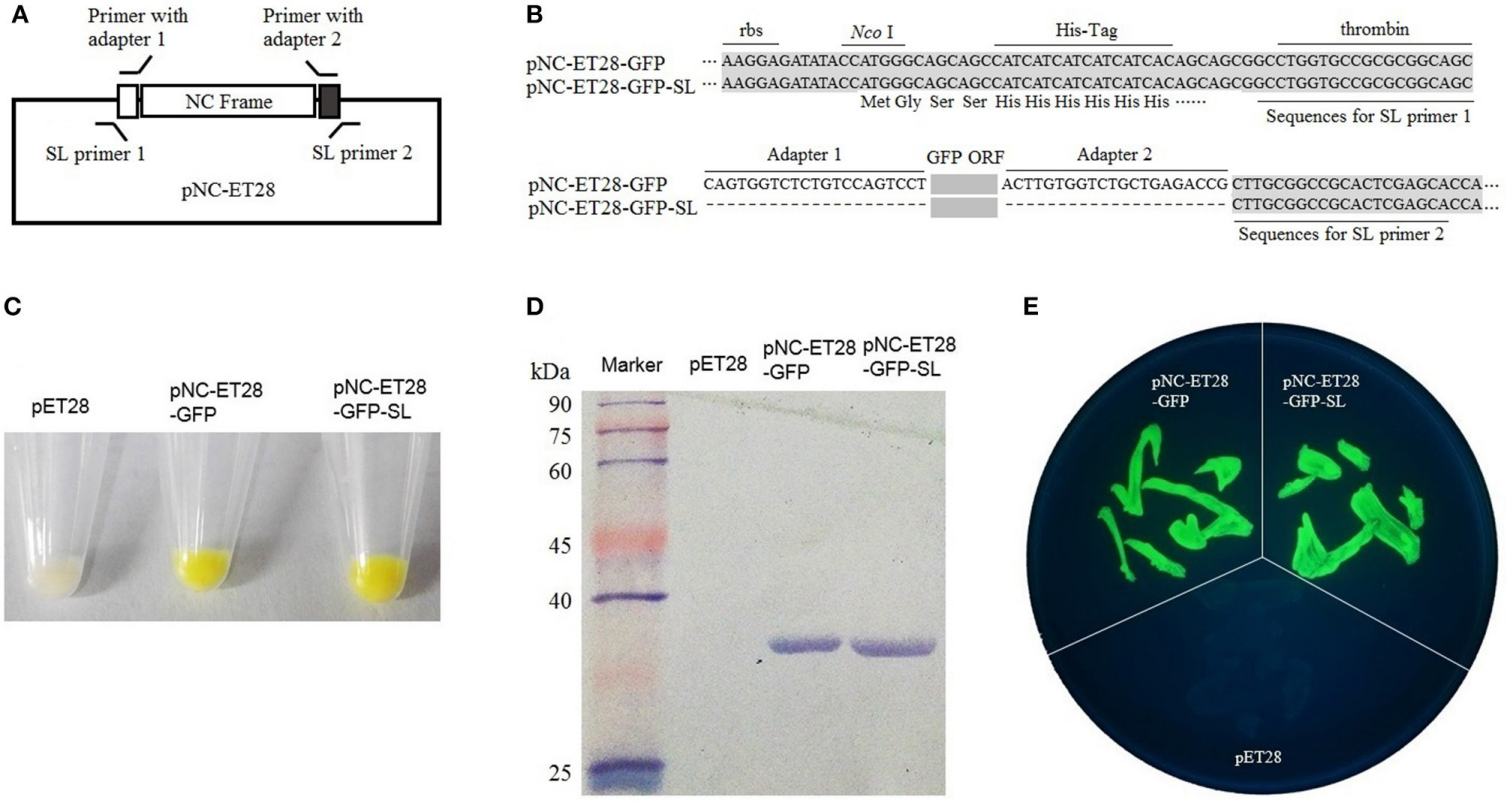
Nimble Cloning was applied for a prokaryotic expression system. **(A)** Schematic of the Nimble Cloning system vector pNC-ET28, which is derived from pET28. The primers with the unique adapters as overlapping sequences were applied for standardized cloning, whereas the primers with the sequences outside the adapters as overlapping sequences were applied for scarless (SL) cloning. **(B)** The sequences around the insertion sites of pNC-ET28-GFP and pNC-ET28-GFP-SL. Unique adapters were used for standardized cloning to generate the pNC-ET28-GFP, while the underline sequences outside the adapters were used as the homologous ends for scarless cloning to generate the pNC-ET28-GFP-SL. **(C–E)** The GFP fluorescence associated with the pNC-ET28-GFP, pNC-ET28-GFP-SL, and empty pET28 vector was observed under a bright light **(C)** and a UV lamp **(E)**. Additionally, GFP was detected in a western blot with an anti-GFP antibody **(D)**.

The pNC-ET28 vector, which is derived from pET28, was used for the prokaryotic expression analysis. The GFP gene was cloned into pNC-ET28 by standardized cloning and scarless cloning to form pNC-ET28-GFP and pNC-ET28-GFP-SL, respectively (Figures 3A,B). The plasmids were transformed into *E. coli* cells, after which GFP fluorescence was observed under a bright light (Figure 3C) and a UV lamp (Figure 3E). Additionally, GFP was detected in a western blot involving the anti-GFP antibody (Figure 3D). There were no differences between pNC-ET28-GFP and pNC-ET28-GFP-SL regarding the resulting protein abundance and activity.

**Figure 4:**
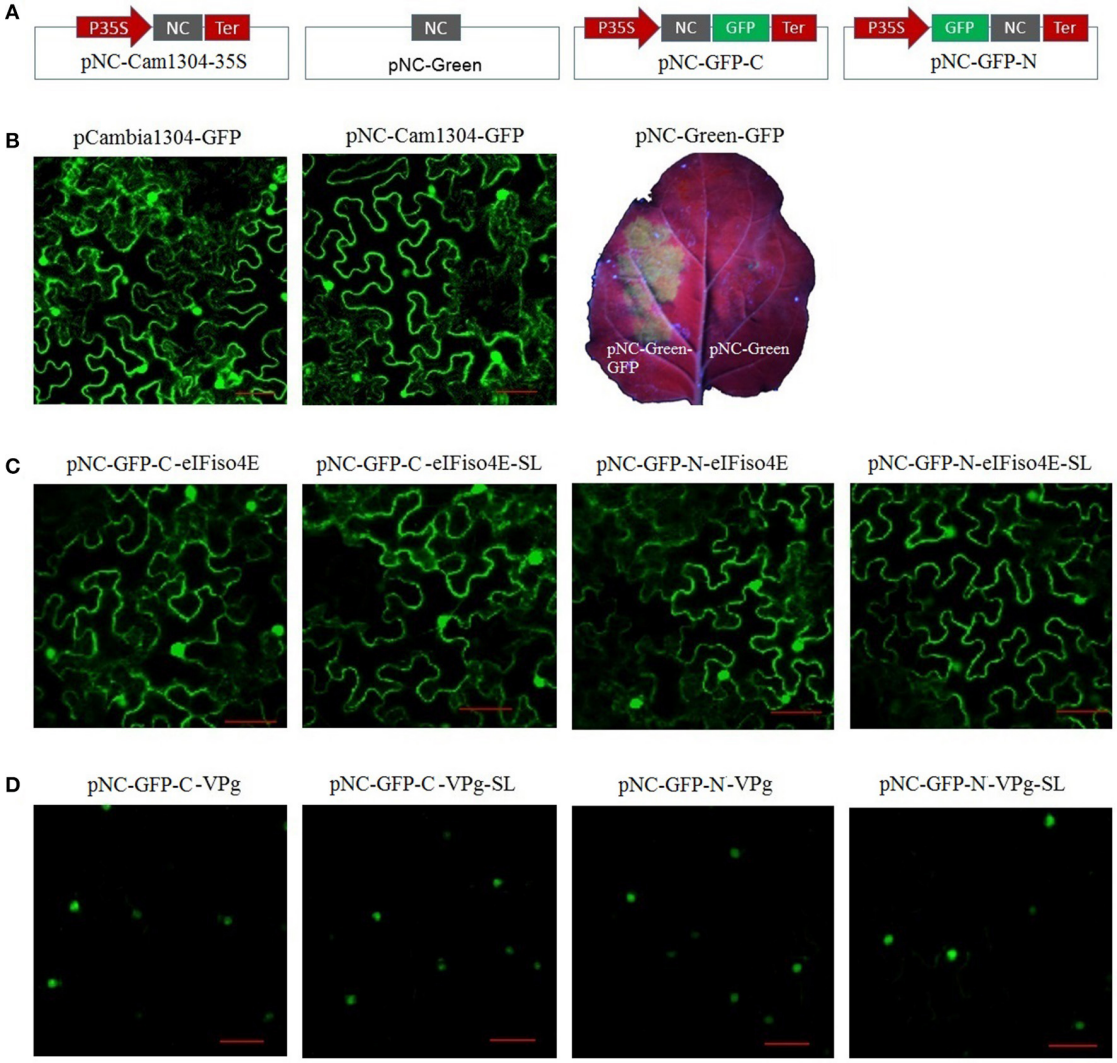
Nimble Cloning was applied for a plant expression system. **(A)** Schematic of the Nimble Cloning system vectors used for gene overexpression in plants. NC, NC frame for Nimble Cloning. **(B–D)** The GFP signal in *Nicotiana benthamiana* leaves was observed with a confocal microscope or a UV lamp. **(B)** Comparison of the GFP signal associated with pCAMBIA1304-GFP and pNC-Cam1304-GFP, and the expression of pNC-Green-GFP. Comparison of the expression of papaya eIFiso4E **(C)** and prsv-VPg **(D)** resulting from different sublocation vectors of the Nimble Cloning system with or without (SL) unique adapters. Papaya eIFiso4E is present in the nucleus and cytoplasm, whereas prsv-VPg is present in the nucleus. Scale bars = 50 μm.

For the plant expression analysis, strong GFP fluorescence was observed for plants regardless of whether they were transformed with pCAMBIA1304-GFP or pNC-Cam1304-GFP, which were constructed by Gibson assembly and standardized Nimble Cloning, respectively, with no significant difference between the two plasmids (Figures 4A,B). The pNC-Green-GFP plasmid constructed by Nimble Cloning for multiple fragments (35S promotor, GFP, and Tnos) also resulted in a strong GFP signal in *N. benthamiana* leaves (Figures 4A,B). Fusion protein production was assessed with sublocation vectors. The papaya *eIFiso4E* gene, which encodes a protein that is present in the nucleus and cytoplasm, as well as the papaya ringspot virus *VPg* gene, which encodes a protein (prsv-VPg) present in the nucleus, were cloned into the sublocation vectors at the N- and C-termini of the GFP gene with the standard and scarless cloning methods of the Nimble Cloning system (Figures 4A,C,D). All of the sublocation constructs were expressed in *N. benthamiana* leaves, and there were no differences between the constructs resulting from the standardized and scarless cloning methods.

### Nimble Cloning for Multiple-Site Cloning

**Figure 5:**
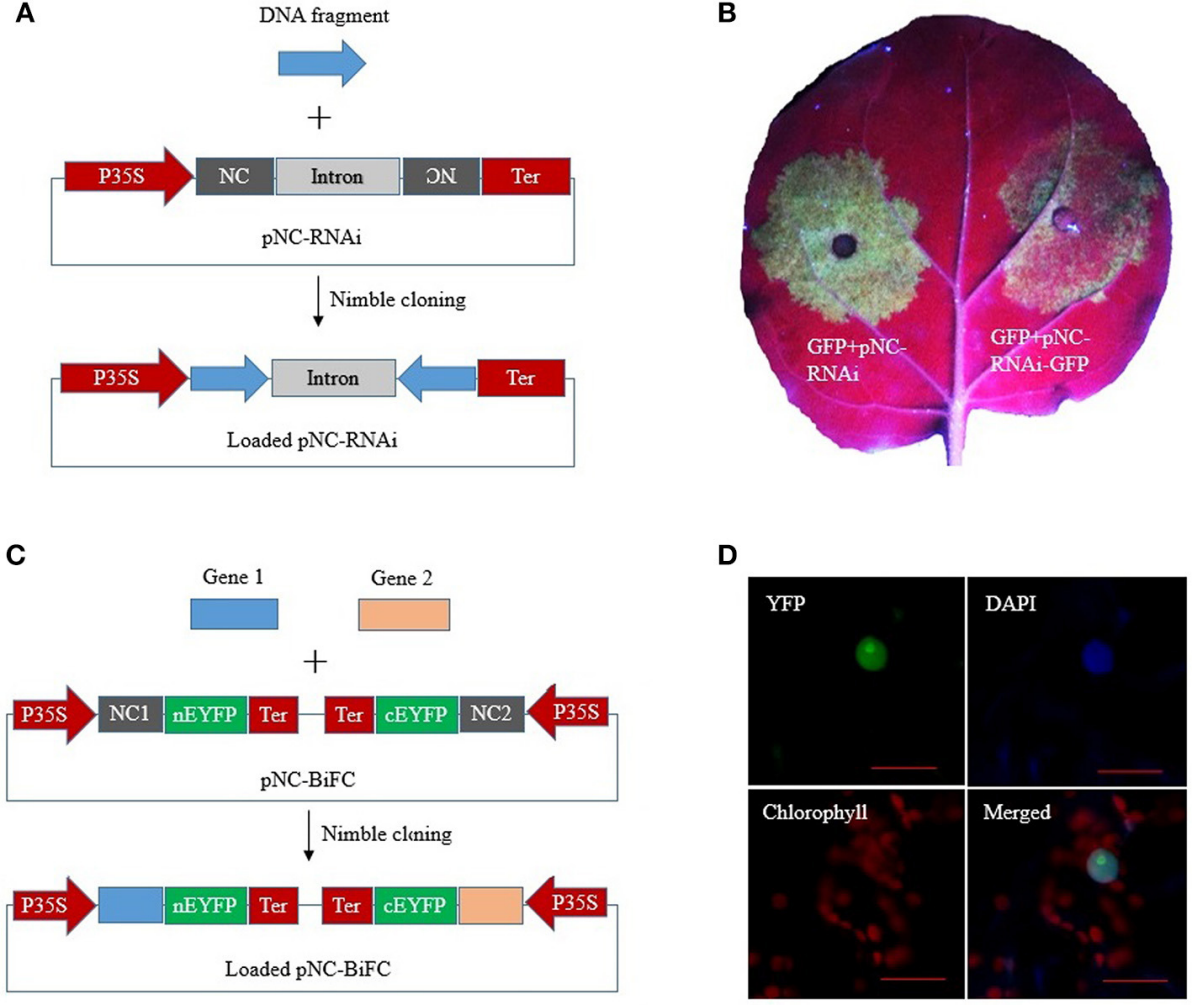
Nimble Cloning for multiple-site cloning in plant RNAi and BiFC. **(A)** Schematic of hpRNA construction by a one-step Nimble Cloning method. A single DNA fragment can be simultaneously cloned into the pNC-RNAi vector in the sense and antisense orientations. **(B)** Silencing of the GFP gene. *Agrobacterium tumefaciens* cells containing pBIN19-GFP were mixed with *A. tumefaciens* cells containing an empty pNC-RNAi vector (left) or the hpRNA construct pNC-RNAi-GFP (right). The cell mixtures infiltrated different parts of the same *Nicotiana benthamiana* leaves. **(C)** Schematic of the construction of a double ORF expression BiFC vector by Nimble Cloning. Two genes can be cloned into the vector in a single reaction. NC 1, NC frame 1; NC 2, NC frame 2. **(D)** The double ORF expression BiFC vector containing the genes encoding papaya eIFiso4E and prsv-VPg was infiltrated into *N. benthamiana* leaves. Fluorescence was observed by confocal microscopy. Scale bars = 20 μm.

RNA interference (RNAi) constructs contain inverted DNA repeats that express self-complementary RNA sequences. We constructed a plant RNAi vector, pNC-RNAi, consisting of an “NC frame–Pdk intron–NC frame” cassette between the CaMV 35S promoter and nos terminator of pCAMBIA1304. The second NC frame was inverted, which enabled a single PCR product amplified with primers containing the unique adapter sequences to be simultaneously recombined into the vector in the sense and antisense orientations to form RNAi constructs via one-step Nimble Cloning (Figure 5A). A 337-bp fragment of the marker gene *EGFP* was amplified and then ligated to the pNC-RNAi vector with the Nimble Mix during a Nimble Cloning reaction. After the subsequent transformation, 12 colonies were analyzed in a PCR assay to confirm the amplification of the expected band. Six colonies were sequenced, and no errors were detected. We then tested the utility of pNC-RNAi-GFP for gene silencing via *A. tumefaciens*-mediated transient expression. The *A. tumefaciens* cells carrying pBIN19-GFP (Santos-Rosa et al., 2008) were mixed with *A. tumefaciens* cells containing an empty pNC-RNAi vector (control) or the RNAi construct pNC-RNAi-GFP. The cell mixtures then infiltrated different parts of the same *N. benthamiana* leaves. The leaf areas infiltrated with the control cells produced a strong GFP signal at 3 days post-agroinfiltration, whereas considerably weaker signals were detected for the leaf areas infiltrated with the cells carrying the RNAi construct (Figure 5B).

A double ORF expression BiFC (bimolecular fluorescence complementation) system enables the coexpression of two fluorescent protein fragments from a single vector (Grefen and Blatt, 2012; Gookin and Assmann, 2014). The double ORF expression vector (pNC-BiFC) we constructed for a plant BiFC assay consisted of two NC frames with different adapters. Two genes of interest flanked by the adapters were simultaneously recombined into the vector by Nimble Cloning (Figure 5C). The genes encoding eIFiso4E and prsv-Vpg were amplified with primers containing the adapters in NC frames 1 and 2, respectively. The PCR products were inserted into the pNC-BiFC vector during a Nimble Cloning reaction. A PCR analysis of the transformants revealed that all 12 tested colonies produced the expected band, with no sequence errors in the six colonies analyzed by sequencing. An *A. tumefaciens*-mediated transient expression experiment was used to evaluate the expression of the plasmid sequences. The *A. tumefaciens* cells containing pNC-BiFC-eIFiso4E/Vpg infiltrated *N. benthamiana* leaves, after which the infiltration areas were observed by confocal microscopy at 3 days post-agroinfiltration. The EYFP fluorescent signal was observed exclusively in the nucleus (Figure 5D). Additionally, fluorescence was undetectable for the negative control leaves that were infiltrated with cells carrying the empty pNC-BiFC vector (data not shown).

## Discussion

### The Advantages of Nimble Cloning Over Other Methods

We herein describe the development of a novel method, Nimble Cloning, which is useful for simple and standardized molecular cloning. This new method has several advantages over the widely used Gibson assembly and Gateway cloning techniques. First, unlike Gibson assembly, which requires an additional step to linearize the destination vector, Nimble Cloning uses circular plasmids. The *Sfi*I and T5 exonuclease enzymes in Nimble Mix simultaneously mediate the linearization of the vector and the generation of 3′-overhangs. Second, Gibson assembly currently lacks a standard primer design method. The Nimble Cloning system involves unique nucleotide sequences (adapters) for standardized cloning, enabling a DNA sequence flanked by the adapters to be cloned into any Nimble Cloning vector. Third, Gibson assembly is limited to PCR products as inserts, and Gateway cloning requires entry clones. In contrast, Nimble Cloning is more flexible because it is appropriate for both PCR products and entry clones. Fourth, Gateway cloning leaves a 25-bp scar sequence at the recombination site, while Nimble Cloning can be applied for scarless cloning, if necessary. T5 exonuclease is not only an exonuclease, but also a flap endonuclease (AlMalki et al., 2016; Xia et al., 2018). It can degrade linear dsDNA or ssDNA by the activity of dsDNA specific exonuclease and ssDNA endonuclease. The *Sfi*I sequence as well as the 21-bp adapters can be degraded by T5 exonuclease for scarless cloning in Nimble Cloning. Finally, Nimble Cloning is relatively inexpensive because it only requires *Sfi*I and T5 exonuclease. These advantages may make Nimble Cloning an ideal system for molecular cloning, especially for researchers in developing countries.

The recombination mechanism underlying the Nimble Cloning system is similar to that of Gibson assembly, and is mainly based on the T5 exonuclease activity that yields the single-stranded DNA overhangs. The main difference between these two methods is that Gibson assembly requires a linearized destination vector, while Nimble Cloning uses circular plasmids. This is because Nimble Cloning involves a mixture of *Sfi*I and T5 exonuclease, which mediate the linearization of the vector and the recombination reaction at the same time. Our results indicated that Nimble Cloning is not only more convenient than Gibson assembly, but it also results in a higher cloning efficiency (Figure 2A). Previous studies confirmed that Golden Gate cloning, which also involves a one-step digestion/ligation reaction, is more efficient than traditional cloning methods requiring a two-step digestion and ligation reaction (Engler et al., 2009; Hsu and Smanski, 2018). The increased efficiency of one-step cloning may be due to the decrease in the number of steps involving the DNA. The manipulation and modification of DNA (e.g., digestion, extraction, column purification, and buffer exchange) are likely to result in DNA damages and a decrease in DNA yield (Engler et al., 2009). The Nimble Cloning vectors are not pre-digested, but are simply added to the reaction mix without the need for a purification step. In addition, removing the Phusion and dNTPs also resulted in increased efficiency (Figure 2A) (Xia et al., 2018).

Both Nimble Cloning and Golden Gate cloning include restriction enzymes in their reaction mixes. Golden Gate cloning uses type IIs restriction enzymes, such as BsaI and BpiI, whereas Nimble Cloning applies a relatively rare-cutting restriction enzyme, *Sfi*I. Specifically, the *Sfi*I recognition sites occur at low frequencies in the *A. thaliana* genome, with only three sites per million base pairs, which is in contrast to the 265 BsaI sites per million base pairs (Ghareeb et al., 2016). This provides a clear advantage of Nimble Cloning over Golden Gate cloning. Furthermore, if there are naturally occurring restriction sites in the DNA fragment of interest, Golden Gate cloning often requires site-directed mutagenesis to eliminate them, which is laborious and possibly ineffective. With Nimble cloning, this issue can be circumvented by first linearizing the destination vector with *Sfi*I followed by the steps of Gibson assembly to clone the DNA fragments of interest. Thus, the occasional presence of the *Sfi*I recognition site will not limit the utility of Nimble Cloning.

### The Unique Nucleotide Sequences and System Vectors Enabled Standardized Molecular Cloning

*In vitro* recombination methods based on short homologous ends, including Gibson assembly and In-Fusion, are becoming popular in molecular cloning research. These methods are mostly sequence-independent, and because of their flexibility, a standardized method for designing the overlapping sequences has not been proposed. To facilitate standardized molecular cloning, two unique nucleotide sequences were designed for the Nimble Cloning system. All of the entry and destination vectors of this system contain unique nucleotide sequences. A PCR product flanked by the unique nucleotide sequences can be cloned into any entry or destination vector. We also evaluated the cloning efficiency and the effects of the unique nucleotide sequences on gene expression. We observed that these sequences resulted in highly efficient cloning (Figures 2A,B), and were suitable for gene expression (Figures 3, 4). Thus, these unique sequences may be useful as standard homologous arms (primers) for *in vitro* recombination-based cloning.

Like Gateway cloning and Golden Gate cloning, Nimble Cloning requires system vectors. We constructed two entry vectors, with ampicillin and kanamycin resistance genes. These entry vectors satisfy the requirements for the cloning of a single fragment. For the cloning of multiple fragments, additional entry vectors will need to be constructed, or the PCR fragments for cloning will need to be used directly. We also constructed more than 40 Nimble Cloning destination vectors, some of which were included in this study. Since there are currently hundreds of expression vectors available for molecular biology research, a viable Nimble Cloning system will require the development of many additional destination vectors. To construct these destination vectors, the NC frame will need to be inserted in the cloning sites (Supplementary Methods S1). Thus, the construction of new destination vectors is simple, and will not limit the application of the Nimble Cloning system. Furthermore, entry and destination vectors form the basis of standardized molecular cloning.

### Nimble Cloning May Facilitate Modular DNA Assembly and Multi-Level Construct Assembly

In addition to standardization, modularity is another principle associated with DNA assembly. The main methods currently used for modular DNA assembly are Golden Gate cloning and Gibson assembly. Golden Gate-based methods, including MoClo (Weber et al., 2011), GoldenBraid (Sarrion-Perdigones et al., 2011; Vazquez-Vilar et al., 2017), and MetClo (Lin and O'Callaghan, 2018), have been applied to construct complex DNA constructs. Gibson-based methods involve unique nucleotide sequences to facilitate the assembly of sequences in the correct order (Casini et al., 2014; Torella et al., 2014a,b; Halleran et al., 2018). However, Gibson-based methods require linearized DNA modules produced via PCR amplification or enzyme digestion in each assembly level. Nimble Cloning enables the direct use of an entry clone for highly efficient cloning, thereby facilitating modular DNA assembly and multi-level construct assembly.

In summary, Nimble Cloning is simple, versatile, and efficient, and is a promising system for the standardized molecular cloning required in diverse applications. The plant expression vectors of pNC system will facilitate the analysis of gene overexpression, protein localization, gene silencing, and protein-protein interactions in plant functional genomics.

## Accession Numbers

Sequence data from this article can be found in the GenBank data libraries under accession numbers: pNC-UC, MK720605; pNC-ET28, MK720606; pNC-Cam1304-35S, MK896896; pNC-Green, MK896901; pNC-GFP-C, MK896905; pNC-GFP-N, MK896906; pNC-RNAi, MK896898; pNC-BiFC, MK896893; pNC-KEn, MN604399; pNC-AEn, MN604400.

## Data Availability Statement

The datasets generated for this study can be found in the GenBank Nucleotide Database with accession numbers MK720605 for pNC-UC and MK720606 for pNC-ET28.

## Author Contributions

PY and PZ conceived the project and designed experiments. PY, YZ, DT, and XL performed experiments. PY, DT, and WS analysis the data. PY wrote the manuscript. All authors commented on the manuscript.

### Conflict of Interest

The authors declare that the research was conducted in the absence of any commercial or financial relationships that could be construed as a potential conflict of interest.

## Supplementary Material

Supplementary Figure S1 Maps of plasmids used in this study. **(A,B)** The prokaryotic expression vectors pNC-UC and pNC-ET28. **(C,D)** The plant expression vector pNC-Cam1304 and pNC-Green. **(E,F)** The plant subcellular location vectors pNC-SubC and pNC-SubN. **(G)** The plant RNAi vector pNC-RNAi. **(H)** The double ORF expression BiFC vector pNC-BiFC.

Supplementary Table S1 Primer sequences used in this study.

Supplementary Methods S1 Standardized molecular cloning by Nimble Cloning.
