# Supplementary figures and images for: Nimble Cloning: A Simple, Versatile, and Efficient System for Standardized Molecular Cloning
Source: Front Bioeng Biotechnol. 2020 Jan 15;7:460. doi: 10.3389/fbioe.2019.00460 (PMC6974442; doi:10.3389/fbioe.2019.00460)

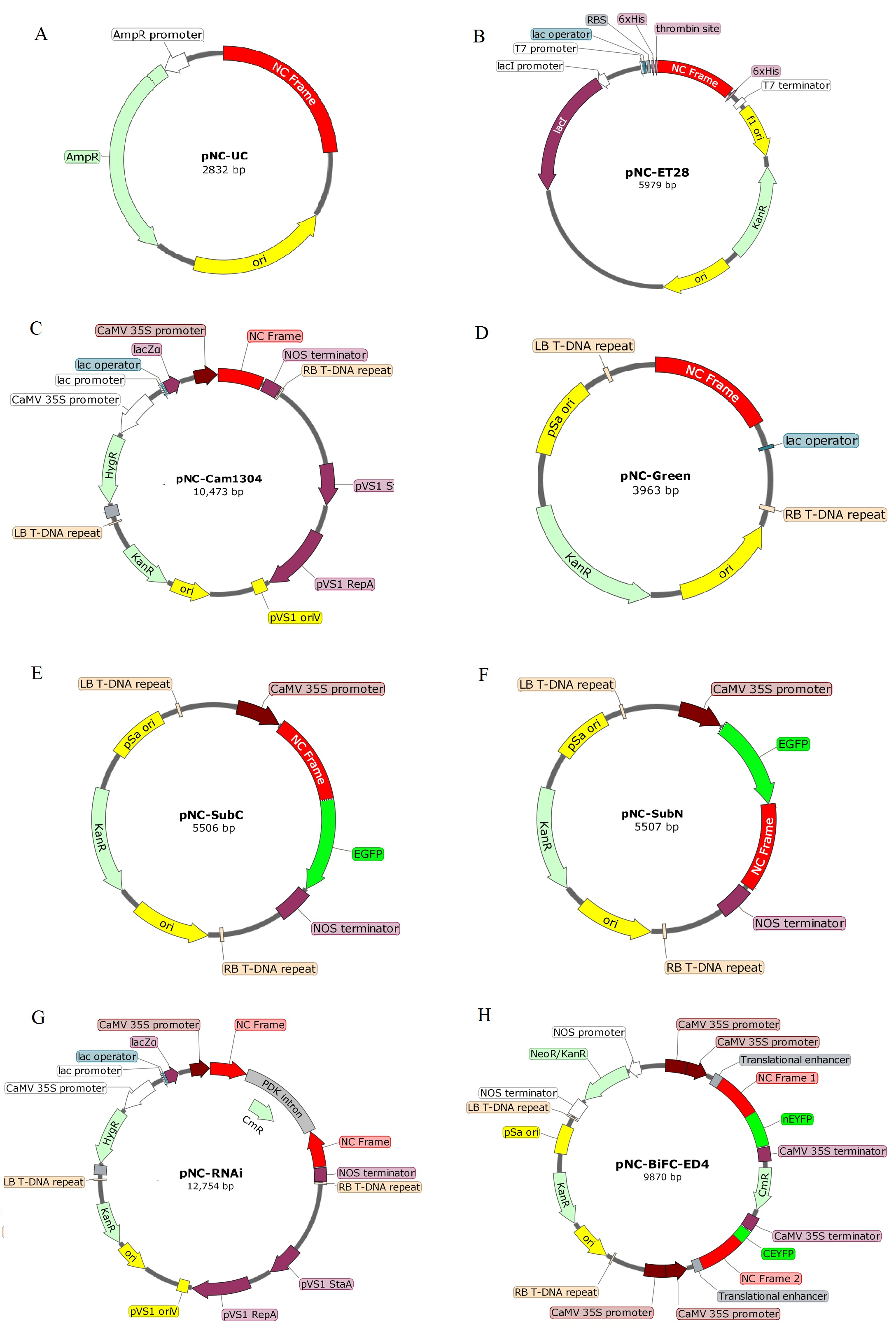

Supplement: Supplementary Figure S1 — Maps of plasmids used in this study. (A,B) The prokaryotic expression vectors pNC-UC and pNC-ET28. (C,D) The plant expression vector pNC-Cam1304 and pNC-Green. (E,F) The plant subcellular location vectors pNC-SubC and pNC-SubN. (G) The plant RNAi vector pNC-RNAi. (H) The double ORF expression BiFC vector pNC-BiFC. [file Image_1.TIF]
