# Supplementary material for: Nimble Cloning: A Simple, Versatile, and Efficient System for Standardized Molecular Cloning
Source: Front Bioeng Biotechnol. 2020 Jan 15;7:460. doi: 10.3389/fbioe.2019.00460 (PMC6974442; doi:10.3389/fbioe.2019.00460)
Supplement: Supplementary Table S1 — Primer sequences used in this study. [file Table_2.DOCX]

Table S1. Primer sequences used in this study

| Primer | | Sequence (5′-3′) | Description |
| --- | --- | --- | --- |
| NC-F | cagtggtctctgtccagtcctggcctcgtcggccattctcgactaagttggcag | | To amplify the NC Frame |
| NC-R | cggtctcagcagaccacaagtggccagactggccctgtgtataagggagcctgac | |  |
| Entry-F | gcctcgtcggccagtggtctctgtccagtcctgacatggattctcgactaagttggcag | | To amplify the cloning |
| Entry-R | ccagactggccggtctcagcagaccacaagttcactggctgtgtataagggagcctgac | | cassette of entry vector |
| UCF | acttgtggtctgctgagacctcactgcccgctttccagtc | | To amplify the backbone of |
| UCR | aggactggacagagaccacttgtgaccgtctccgggagct | | pUC19 for pNC-UC |
| NC-ET28F | cctggtgccgcgcggcagccagtggtctctgtccagtcct | | To amplify the NC Frame for |
| NC-ET28R | tgctcgagtgcggccgcaagcggtctcagcagaccacaagt | | pNC-ET28 |
| NC-CamF | gaacacgggggactcttgaccagtggtctctgtccagtcct | | To amplify the NC Frame for |
| NC-CamR | gagctggtcaccaattcacacggtctcagcagaccacaagt | | pNC-Cam1304 |
| NC-SubCF | gaacacgggggactcttgacagtggtctctgtccagtcct | | To amplify the NC Frame for |
| NC-SubCR | cctcgcccttgctcaccatcggtctcagcagaccacaagt | | pNC-SubC |
| SubC-GFPF | gatggtgagcaagggcgaggag | | To amplify the GFP for |
| SubC-GFPR | ggaaattcgagctggtcacctcacttgtacagctcgtccatgc | | pNC-SubC |
| SubN-GFPF | gaacacgggggactcttgacatggtgagcaagggcgaggag | | To amplify the GFP for |
| SubN-GFPR | cttgtacagctcgtccatgc | | pNC-SubN |
| NC-SubNF | gcatggacgagctgtacaagcagtggtctctgtccagtcct | | To amplify the NC Frame for |
| NC-SubNR | ggaaattcgagctggtcacctcacggtctcagcagaccacaagt | | pNC-Subn |
| RNAi-NCF | gaacacgggggactcttgacagtggtctctgtccagtcct | | To amplify the NC Frame for |
| RNAi-NCR | tttccttaccaagggctcgtggtctcagcagaccacaagt | | pNC-RNAi |
| Intron-F | ccacgagcccttggtaaggaaat | | To amplify the PDK intron for |
| Intron-R | ccagaccaactgtaatcaatcc | | pNC-RNAi |
| RNAi-INCF | ggaaattcgagctggtcaccagtggtctctgtccagtcct | | To amplify the inverted NC |
| RNAi-INCR | tggattgattacagttggtctggtctcagcagaccacaagt | | Frame for pNC-RNAi |
| NYFP-F | acttgtggtctgctgagacccatggtgagcaagggcgagg | | To amplify the NYFP for |
| NYFP-R | aggactggacagagaccactggctatcgttcgtaaatggtg | | pNC- BiFC |
| CYFP-F | agacctggatgtttccagtggggcagcgtgcagctcgccg | | To amplify the CYFP for |
| CYFP-R | acgcatggtcgagccaactct gctatcgttcgtaaatggtg | | pNC- BiFC |
| 35S-F | cgaggtcgacggtatcgataagcttgttgtaaaacgacggccagt | | To amplify the expression |
| Ter-R | tagtggatcccccgggctgcaggaattcgcgtcactggattttggttttag | | cassettes for pNC- BiFC |
| NC2-F | gagttggctcgaccatgcgtggcctcgtcggccattctc | | To amplify the NC Frame 2 |
| NC2-R | ccactggaaacatccaggtctggccagactggccctgtgt | | for pNC- BiFC |
| cm-F | ctaaaaccaaaatccagtgacgcttatgggaccgacatatcag | | To amplify chloramphenicol |
| cm-R | ctaaaaccaaaatccagtgacgcccggtaagaggttccaactt | | resistant gene for pNC- BiFC |
| LacZ-F | agtggtctctgtccagtcctgcgcaacgcaattaatgtgag | | For standard cloning of LacZ |
| LacZ-R | ggtctcagcagaccacaagtacagcttgtctgtaagcgga | |  |
| LacZ-SL-F | agctcccggagacggtcacagcgcaacgcaattaatgtgag | | For scarless cloning of LacZ |
| LacZ-SL-R | gactggaaagcgggcagtgaagtacagcttgtctgtaagcgga | |  |
| GFP-F | agtggtctctgtccagtcctatggtgagcaagggcgaggagct | | For standard cloning of GFP to |
| GFP-R | ggtctcagcagaccacaagttcacttgtacagctcgtccatgc | | pNC-ET and pNC-Cam1304 |
| GFP-SL-F | gcctggtgccgcgcggcagcatggtgagcaagggcgaggagct | | For scarless cloning of GFP |
| GFP-SL-R | tgctcgagtgcggccgcaagtcacttgtacagctcgtccatgc | | into pNC-ET |
| Gib- GFP-F | gaacacgggggactcttgacatggtgagcaagggcgaggagct | | For cloning of GFP into |
| Gib- GFP-R | gagctggtcaccaattcacactcacttgtacagctcgtccatgc | | pCambia1304 by Gibson |
| 35S-F | agtggtctctgtccagtcctcatggagtcaaagattcaaatagag | | For the three fragments cloning |
| 35S-R | cactggaaacatcaaggtcgagtcccccgtgttctctcca | | of 35S, GFP and Ter into |
| 3F- GFP-F | cgaccttgatgtttccagtgatggtgagcaagggcgaggagct | | pNC-UC and pNC-Green |
| 3F- GFP-R | acgcaaagtcgagccaactctcacttgtacagctcgtccatgc | |  |
| Ter-F | gagttggctcgactttgcgtcgttcaaacatttggcaataaag | |  |
| Ter-R | ggtctcagcagaccacaagtcccgatctagtaacatagatg | |  |
| iso-F | agtggtctctgtccagtcctatggcgagtgaggtagcgat | | For the standard cloning of |
| iso-R | ggtctcagcagaccacaagtcacattgtatcgacttttttgc | | papaya eIFiso4E |
| iso-subc-SL-F | agaacacgggggactcttgaatggcgagtgaggtagcgat | | For scarless cloning of |
| iso-subc-SL-R | tcctcgcccttgctcaccatcacattgtatcgacttttttgc | | eIFiso4E into pNC-SubC |
| iso-subn-SL-F | gcatggacgagctgtacaagatggcgagtgaggtagcgat | | For scarless cloning of |
| iso-subn-SL-R | aattcgagctggtcacctcacacattgtatcgacttttttgc | | eIFiso4E into pNC-SubN |
| VPg-F | agtggtctctgtccagtcctatgggtttctccgcgcgacag | | For the standard cloning of |
| VPg-R | ggtctcagcagaccacaagtttcatggtgaacagattttgtt | | prsv-VPg |
| VPg-subc-SL-F | agaacacgggggactcttgaatgggtttctccgcgcgacag | | For scarless cloning of |
| VPg-subc-SL-R | tcctcgcccttgctcaccatttcatggtgaacagattttgtt | | prsv-VPg into pNC-SubC |
| VPg-subn-SL-F | gcatggacgagctgtacaagatgggtttctccgcgcgacag | | For scarless cloning of |
| VPg-subn-SL-R | aattcgagctggtcacctcattcatggtgaacagattttgtt | | prsv-VPg into pNC-SubN |
| RNAi-GFP-F | agtggtctctgtccagtccttgaaccgcatcgagctgaag | | For the cloning of a part of |
| RNAi-GFP-R | ggtctcagcagaccacaagtgaccatgtgatcgcgcttct | | GFP into pNC-RNAi |
| VPg-NC2-F | gagttggctcgaccatgcgtatgggtttctccgcgcgacag | | For the cloning of prsv-VPg |
| VPg-NC2-R | cactggaaacatccaggtctttcatggtgaacagattttgtt | | into NC2 of pNC-BiFC |
