# Supplementary material for: Nimble Cloning: A Simple, Versatile, and Efficient System for Standardized Molecular Cloning
Source: Front Bioeng Biotechnol. 2020 Jan 15;7:460. doi: 10.3389/fbioe.2019.00460 (PMC6974442; doi:10.3389/fbioe.2019.00460)
Supplement: Supplementary Methods S1 — Standardized molecular cloning by Nimble Cloning. [file Table_1.DOCX]

Standardized molecular cloning by Nimble Cloning

1. Primer design

Forward primer: 5′- agtggtctctgtccagtcct- gene specific forward primer-3′

Reverse primer: 5′- ggtctcagcagaccacaagt- gene specific reverse primer-3′

agtggtctctgtccagtcct and ggtctcagcagaccacaagt are the two unique nucleotide sequences (adapters) for standardized cloning. A DNA sequence flanked by the adapters can be cloned into any Nimble Cloning vector, including entry vector and destination vector.

2. Preparation of destination vector

The destination vectors of Nimble Cloning system contain the NC frame. To construct a new destination vectors, the NC frame needs to be inserted in the cloning sites. The primers sequences for amplifying the NC frame is:

Forward primer: 5′- cagtggtctctgtccagtcct- 3′

Reverse primer: 5′- cggtctcagcagaccacaagt-3′

Gibson assembly is recommended to insert the NC frame into the cloning sites. The destination vectors should be free of SfiI sites, except for the NC frame. If SfiI sites present in the destination vector, they should be eliminated by site-directed mutagenesis.

The circular plasmid of destination vector can be directly used for Nimble Cloning reaction.

3. PCR and purification

The PCR for the gene of interest is carried under normal reaction conditions and the product is purified by Gel purification kit. The purified product can be directly used for Nimble Cloning reaction, or be cloned into entry vector by Gibson assembly.

4. Nimble Cloning reaction

Destination vector 1-4 μl (20-100 ng)

PCR product or entry clone 1-4 μl (10-50 ng)

Sterile water to 5 μl

Nimble Mix 5 μl

Total 10 μl

Incubate at 50°C for 1 h. The reaction mixture can be subsequently used for a transformation or stored at −20 °C if not immediately used.

If the PCR product contains SfiI site, the destination vector should be digested by SfiI, then used for Gibson assembly (two-step cloning).

5. Transformation

2-10 μl reaction mixture is transformed into 50-100 μl *E. coli* competent Cell with normal transformation procedure.
